# Rechazo y gestión en vacunaciones: sus claroscuros

**DOI:** 10.26633/RPSP.2019.54

**Published:** 2019-06-07

**Authors:** María Hortal, José Luis Di Fabio

**Affiliations:** 1 Universidad de la República Programa de Desarrollo de las Ciencias Básicas Uruguay Programa de Desarrollo de las Ciencias Básicas, Universidad de la República, Uruguay.; 2 Consultor internacional Consultor internacional Consultor internacional.

**Keywords:** Inmunización, negativa a la vacunación, programas de inmunización, Immunization, vaccination refusal, immunization programs, Imunização, recusa de vacinação, programas de imunização

## Abstract

Durante muchos años, vacunar a los niños era una tradición incuestionable; sin embargo, en la actualidad hay gran preocupación por el creciente rechazo a las vacunas de la infancia, además de otros obstáculos que no son fáciles de visualizar y que afectan las coberturas por vacunación.

Se señalan múltiples factores intervinientes en los rechazos a una vacuna o a la vacunación en general por la acción de grupos antivacunas y por la desinformación o divulgación de datos erróneos. En algunos países, se agregan atrasos en el cumplimiento del esquema de inmunizaciones por posibles fallas en la gestión de los programas. Todo esto compromete el nivel efectivo de las coberturas de vacunación y constituye una seria amenaza para la salud pública.

Las poblaciones susceptibles se modifican de manera constante por cambios epidemiológicos determinados por fenómenos como la globalización o diferentes conflictos que interfieren el accionar sanitario. En época reciente, se han registrado brotes de enfermedades antes controladas como la difteria, la tos ferina y el sarampión, ya sea por casos importados o por fallas en los programas nacionales de inmunización.

Se intentó explorar distintos componentes que se suman a la frecuencia creciente de los rechazos a las vacunas, lo que obliga a una revisión de las causas y al diseño de estrategias y enfoques innovadores para recuperar la aceptación de la vacunación y su lugar como la herramienta más costo-efectiva en salud pública.

## Abstract (en)

Vaccinating children has been an unquestioned tradition for many years. However, there is now great concern over the growing rejection of childhood vaccination, as well as other less evident obstacles that affect vaccination coverage.

Multiple factors are involved in the rejection of a specific vaccine or vaccination in general, including actions by anti-vaccination groups, as well as disinformation or the dissemination of erroneous information. In some countries, delays in completing the immunization schedule may be due to poor program management. These factors compromise effective vaccination coverage, constituting a serious threat to public health.

Susceptible populations constantly change, due to epidemiological shifts determined by phenomena such as globalization and various conflicts that interfere in the operation of health services. In recent years there have been outbreaks of previously controlled diseases such as diphtheria, whooping cough, and measles, resulting both from imported cases and from deficiencies in national immunization programs.

This paper explores different aspects of the increasing frequency of vaccine rejection. There is a need for a review of its causes and for the design of innovative strategies and approaches to regain acceptance of vaccination and its place as the most cost-effective tool in public health.

## RESUMO

Durante muitos anos, vacinar os filhos foi uma tradição inquestionável; atualmente, porém, há grande preocupação com a crescente recusa das vacinas da infância, além de outros obstáculos mais difíceis de visualizar que afetam a cobertura vacinal.

Múltiplos fatores concorrem para influenciar a recusa a uma vacina específica ou à vacinação em geral, seja pela ação de grupos antivacina ou pela desinformação ou divulgação de dados incorretos. Em alguns países, a situação é complicada por atrasos no cumprimento do calendário vacinal devido a possíveis falhas na gestão dos programas. Tudo isso compromete o nível efetivo das coberturas de vacinação e constitui grave ameaça à saúde pública.

As populações suscetíveis a doenças imunopreveníveis estão em constante mutação devido a mudanças epidemiológicas, determinadas por fenômenos como a globalização ou diferentes conflitos que interferem com as ações de saúde. Recentemente, foram registrados surtos de doenças antes controladas, como a difteria, a coqueluche e o sarampo, seja por casos importados ou por falhas nos programas nacionais de imunização.

Este artigo visa explorar os diferentes componentes que contribuem para a frequência crescente da recusa vacinal, o que exige uma reanálise dos fatores causais e o delineamento de estratégias e enfoques inovadores para recuperar a aceitação das vacinas e de seu papel como a mais custo-efetiva das ferramentas em saúde pública.

## Antecedentes

Durante muchos años, vacunar a los niños era una tradición incuestionable, pero en la actualidad hay gran preocupación por el creciente rechazo a las vacunas de la infancia, y también otros obstáculos difíciles de visualizar y que afectan las coberturas por vacunación. Vale la pena reflexionar sobre la gran diversidad de situaciones a enfrentar y modificar para alcanzar y mantener los beneficios de las vacunas proclamados por la Organización Mundial de la Salud (OMS) para la década 2010-2020 (1).

Los avances tecnológicos alcanzados en la segunda mitad del siglo XX posibilitaron la producción industrial de vacunas efectivas frente a las principales enfermedades infecciosas de la infancia. Tales logros, sumados a progresos en la higiene ambiental y en la organización sanitaria, permitieron contener la mayoría de las enfermedades infecciosas (2). La instrumentación del Programa Ampliado de Inmunizaciones (PAI) en la Región de las Américas permitió el control o la disminución de la incidencia de la mayoría de las enfermedades infecciosas de la infancia y también prevenir infecciones en adultos (3). Fue así como, en países donde se alcanzaron niveles de coberturas de vacunación elevados en la población expuesta, se fue perdiendo, de manera inadvertida, el temor a las enfermedades controladas por la vacunación. Además, las generaciones jóvenes de médicos y de familias, no piensan en, ni conocen, el impacto del sarampión o de la tos convulsa, entre otros ejemplos, e incluso no tienen idoneidad para diagnosticarlos.

En Europa, al principio los grupos ecologistas, y luego parte de la sociedad civil, pusieron en duda las ventajas de las vacunas cada vez más numerosas y efectivas. Esa inquietud se extendió al resto del mundo como una “enfermedad contagiosa”. De las dudas se pasó al rechazo a la vacunación, en ocasiones a una vacuna en particular, pero con frecuencia en forma global (4). Este revisionismo se traduce en una reducción de los niveles de cobertura con cada vacuna y la posible reaparición de enfermedades olvidadas (5).

A pesar de que la OMS aspiraba a que el período 2010-2020 fuera la “década de la vacunación”, ya en el 2012 los organismos sanitarios internacionales, frente al creciente rechazo a las vacunas, dudaban que se alcanzaran coberturas globales (6). La Organización Panamericana de la Salud (OPS) se hizo eco de las objeciones planteadas y comenzó a indagar sobre influencias individuales o grupales con relación a los programas de vacunación y los determinantes contextuales o los inherentes a las vacunas para elaborar una hoja de ruta con acciones que eviten los rechazos (7).

Ya sea por retraso en o por rechazo hacia la vacunación, en la actualidad nos enfrentamos a un problema complejo y multifactorial, cuya magnitud y componentes debemos analizar si deseamos reducirlo. La reticencia a la vacunación, por retardo o cuestionamientos, varía según los países, lugares y contextos socioculturales de las poblaciones. En consecuencia, resulta imprescindible comprender no solo las motivaciones que intervienen en los atrasos y rechazos, sino también las realidades donde se insertan y la contribución de otros factores, que también son diversos y van desde la calidad de la gestión de los programas de vacunación hasta posibles intereses políticos (8, 9).

En el año 2017, la Comisión Europea, con base en las opiniones contrarias a la vacunación de ciudadanos de muchos de los Estados Miembros, estableció una hoja de ruta con el fin de proporcionar información apropiada para implementar medidas que contrarresten los riesgos de los rechazos. En diciembre del año siguiente, formuló una recomendación para reforzar la cooperación de la Unión Europea en materia de enfermedades prevenibles mediante vacunación, que incluye hacer frente a la reticencia a la vacunación (10).

Si bien a la fecha existen numerosas publicaciones sobre el rechazo a las vacunas, son escasas las comunicaciones latinoamericanas referidas al tema (11). Tampoco se ha intentado una visión global en la que se aúnen las dudas o rechazos con las posibles fallas en la complejidad de los diversos componentes que inciden en los programas de vacunación.

## Rechazo a las vacunas

De persistir el rechazo por parte de la población susceptible y la presencia de obstáculos en cualquier aspecto de los programas nacionales o locales, se alterarán los niveles efectivos de control de las enfermedades inmunoprevenibles. Esto determinará un posible retroceso con registros de morbimortalidad similares a los de principios del siglo XX (12). Tal posibilidad se ha considerado como uno de los diez desafíos prioritarios en materia de salud, que concitan la atención de la OMS y de sus socios en salud en 2019.

Con el tiempo, ha ido en aumento el arsenal de vacunas y su correspondiente efectividad. No obstante, esa ventaja para la salud se acompaña de repetidas inyecciones que son resistidas por los niños a medida que crecen, por eso algunas familias comienzan a cuestionar el valor de las vacunas. Este tipo de vacilaciones se suman a los crecientes movimientos antivacunas. Las objeciones planteadas son muy diversas, desde considerar que las vacunas combinadas son procedimientos artificiales, potencialmente dañinos, hasta negar de plano la necesidad de la vacunación contra el riesgo de una enfermedad infecciosa inexistente.

El temor a reacciones adversas luego de una vacuna, o la aparición de una enfermedad tiempo después en población vacunada, son argumentos frecuentes. Se cuestiona la falta de estudios en el largo plazo que demuestren la inocuidad de las vacunaciones. También se desconfía de las vacunas por ser objeto de lucro por parte de la industria farmacéutica, pero no se tiene en cuenta que esta, a su vez, brinda un servicio importante para la salud, en general no cubierto por una producción nacional. Toda la información a la población por parte de los distintos actores ha de incluir mensajes claros sobre los beneficios de cada nueva vacuna y sobre la vacunación en general.

La oposición a la vacunación provoca un conflicto entre el derecho individual a no vacunarse y la responsabilidad de cada individuo de contribuir a la inmunidad comunitaria o de rebaño. La decisión corresponde a los familiares o responsables de los niños, pero en la toma de esta decisión, en la mayoría de los casos se ignora que, además de inmunizar al niño, se contribuye a una salud colectiva.

Está demostrado que la inmunidad comunitaria reduce los riesgos de la diseminación y trasmisión de agentes infecciosos en el ambiente y evita así posibles brotes epidémicos, ya que se alcanzan niveles de cobertura vacunal relativamente altos. El número de individuos inmunes a una determinada enfermedad en relación con el número de individuos con susceptibilidad específica a la misma enfermedad, se relaciona en forma directa con la posibilidad de que esa enfermedad reaparezca y prospere (13). Cabe preguntarse hasta dónde llegan los derechos individuales o la solidaridad con el conjunto, ya que la negativa puede constituir una amenaza para la salud de muchos. Entonces, la vacunación obligatoria se justificaría como una responsabilidad del Estado y una medida de salud pública.

Esa responsabilidad la comparten también los pediatras, que tienen una influencia decisiva en el cuidado de la salud de los niños y deberán informar a las familias sobre las ventajas ofrecidas por las vacunas, indicarlas en cada oportunidad y procurar que consten en el carné del niño. De la misma forma, es también su responsabilidad informar sobre el impacto negativo que la omisión de vacunar ejerce sobre otros niños.

El movimiento antivacunas emplea diferentes formas de comunicación, desde mensajes orales hasta libros o artículos de divulgación (prensa, televisión, etc.) (14, 15). Las redes sociales tienen una importante penetración en amplios sectores de la población, desde las cuales se difunden mensajes contrarios a las vacunaciones y se crean controversias y confusión (16).

Un ejemplo claro de otros efectos negativos es una publicación científica del año 1998 que se refería a una posible asociación entre la vacuna triple viral de sarampión, rubéola y parotiditis (MMR, por sus siglas en inglés) y el autismo (17). A pesar de múltiples objeciones, el artículo fue publicado y recién fue retirado una década después. El daño que esta publicación y su difusión por los medios de prensa causó en su momento fue muy importante en el Reino Unido (18). Su secuela aún perdura en varios países y es utilizada por los grupos antivacunas (19). Algo similar ocurre con la supuesta aparición del síndrome de Guillen-Barré luego de la aplicación de diferentes vacunas (20).

No menos importantes son las comunicaciones que respaldan el valor de las vacunas, en las que se destacan las consecuencias perjudiciales de los rechazos. Es necesario presentar y traducir la evidencia científica en una forma comprensible para la población en general, con diferentes estrategias según el contexto y la audiencia, de manera de contrarrestar la propagación de información falsa. Es posible incluir herramientas digitales apropiadas y buscar alianzas con la sociedad civil y otras partes interesadas. Se debe trabajar en la formación de actores relevantes como los trabajadores de la salud, los educadores, interlocutores sociales y los medios de comunicación. Estos se transforman en multiplicadores de la información y pueden luchar de manera activa contra la complacencia o la falta de confianza en las inmunizaciones.

La optimización de las coberturas ha de mantenerse en el tiempo, pues aun con programas exitosos siempre quedan individuos susceptibles: queda un porcentaje sin inmunizar por diferencia en las coberturas o por falta de respuesta a los antígenos suministrados. A esa población susceptible se suman las cohortes de nacimientos hasta que alcanzan la edad para recibir las vacunas indicadas.

## Los cambios en las poblaciones humanas

Diferentes poblaciones y etnias, en áreas urbanas o rurales de distintos países, requieren estrategias y recursos apropiados para la implementación de los programas de vacunación.

Los cambios epidemiológicos en las poblaciones a inmunizar tienen gran trascendencia para lograr el control de las infecciones. Los cambios sociales profundos y trascendentales modifican cada vez más la epidemiología en las poblaciones susceptibles. La tendencia fuerte e incoercible a la urbanización facilita el acceso de las poblaciones infantiles susceptibles, y permite evitar las dificultades de las zonas rurales. No obstante, con la acumulación de población, se dan condiciones propicias para una mayor facilidad de contagio de enfermedades infecciosas, por lo que se requiere optimizar las coberturas de las vacunas.

La globalización, con su rápido intercambio mundial de mercaderías y personas, ha eliminado las fronteras entre las naciones. Es así que se identifican casos importados de diferentes enfermedades infecciosas de manera inesperada.

En el 2016, el sarampión se consideraba eliminado de las Américas, pero resurgió a partir de una epidemia en Venezuela, que se diseminó desde Méjico hasta Argentina. Ya en agosto del 2018 había un total de 5 004 casos confirmados de sarampión, con 68 muertes en 11 países de la Región de las Américas. Venezuela reportó a la OPS 3 545 casos de la enfermedad con 62 muertes y, Brasil, 1 237 casos con 6 muertes; los primeros 55 casos registrados correspondieron a niños hijos de emigrantes venezolanos (21).

En época reciente, se iniciaron brotes de sarampión en Nueva York por casos importados, lo que indica que estas situaciones pueden ocurrir en cualquier lugar y oportunidad (22).

Es evidente que los regímenes políticos, la falta de trabajo, la pobreza, el terrorismo y las guerras distorsionan, cada vez con mayor frecuencia, la organización sanitaria en muchos países. En un mundo globalizado, esas dramáticas turbulencias, si bien distantes desde el punto de vista geográfico, pueden alcanzar cualquier lugar. En consecuencia, es crucial para las poblaciones infantiles optimizar su protección con vacunas.

Las migraciones masivas de personas están alterando, con su llegada, a las organizaciones sanitarias locales o nacionales en los diferentes continentes (como ocurre en Europa mediterránea y en América Central) (23). Las poblaciones infantiles desplazadas suelen tener los casos índices de diferentes enfermedades infecciosas (24).

Existen también otros movimientos poblacionales asociados a estos riesgos sanitarios. Muchos grupos humanos deben enfrentarse a cambios ecológicos importantes, como establecerse en áreas recientemente deforestadas o soportar fenómenos climáticos como inundaciones y huracanes, entre otros.

Por otra parte, la prolongación de la vida humana crea cambios demográficos por aumento de la población añosa. Aún no se ha profundizado en la evaluación de la duración de la inmunidad específica con el paso de los años y la posibilidad de que los individuos mayores constituyan un nuevo reservorio para algunos agentes infecciosos.

Los disturbios que causan cambios epidemiológicos a nivel mundial ponen en riesgo conquistas trascendentales logradas con los programas de vacunación. No se trata solo de la erradicación de la viruela, sino de otros éxitos como regiones enteras libres de poliomielitis y la notoria disminución mundial de la rubéola congénita, del tétanos materno y neonatal y del sarampión. Al finalizar el siglo XX, las estadísticas de morbilidad y mortalidad por infecciones inmunoprevenibles habían caído de manera notable, pero los múltiples factores señalan la fragilidad de esos éxitos.

## Reflexiones finales

A la fecha, no se ha imaginado un mundo sin vacunas. Meditar sobre esa posible realidad, sería tal vez, el mejor antídoto para los rechazos. Tampoco se ha pensado lo que sucedería si las empresas proveedoras de vacunas perdieran el interés en desarrollar nuevos recursos y dejaran de producir las vacunas en uso.

La prevención de las enfermedades infecciosas de la infancia constituye una de las conquistas más importantes del siglo XX y de progresos en los años siguientes. Ese avance en la prevención merece toda clase de esfuerzos individuales y colectivos para su preservación. Gran multiplicidad de factores permite mantener esas conquistas, pero, a la vez, las complejidades de los programas de vacunación conllevan una gran fragilidad porque cualquier falla puede comprometer los resultados. A pesar de las conquistas alcanzadas, persisten desafíos para prevenir otras enfermedades infecciosas como el dengue y la malaria entre muchas otras, o mejorar las vacunas existentes mediante diferentes tecnologías y modalidades de administración (25). La creciente frecuencia de los rechazos a las vacunas causa inquietud, pero, a la vez obliga a una revisión de las causales y diseño de estrategias y enfoques innovadores (12, 26) en el actual marco social y político de cada realidad y país para recuperar la aceptación de la vacunación y su lugar como la herramienta más costo-efectiva en salud pública con el fin de alcanzar y superar la meta fijada por OMS para el 2020.

## Contribución de los autores.

Todos los autores colaboraron en la elaboración del manuscrito, revisaron y aprobaron la versión final.

## Declaración.

Las opiniones expresadas en este manuscrito son responsabilidad del autor y no reflejan necesariamente los criterios ni la política de la *RPSP/PAJPH* y/o de la OPS.
